# Organizational capacity to eliminate outcome disparities in addiction health services

**DOI:** 10.1186/1940-0640-10-S1-A19

**Published:** 2015-02-20

**Authors:** Erick G Guerrero, Gregory Aarons, Christine Grella, Bryan R Garner, Benjamin Cook, William A Vega

**Affiliations:** 1 School of Social Work, University of Southern California, Los Angeles, CA, 90089, USA; 2 Department of Psychiatry, University of California, San Diego, San Diego, CA, 92093-0812, USA; 3 Integrated Substance Abuse Programs, Department of Psychiatry & Biobehavioral Sciences, University of California, Los Angeles, Los Angeles, CA, 90025, USA; 4 Chestnut Health Systems, Normal, IL, 61761, USA; 5 Department of Psychiatry, Harvard Medical School, Boston, MA, 02215, USA

## Background

Identifying provider characteristics associated with greater capacity to implement new practices geared toward reducing the disparities gap in health-care services has become a chief priority. Yet, there is limited information on conceptual frameworks and methodologies to understand key organizational factors associated with positive client outcomes.

## Purpose

To evaluate program capacity factors associated with client outcomes in publicly funded substance abuse treatment in one of the most populous and diverse regions of the United States.

## Methods

Using multilevel cross-sectional analyses of program data (n = 97) merged with client data from 2010 to 2011 for adults (n = 8599), we examined the relationships between program capacity (leadership, readiness for change, and Medi-Cal payment acceptance) and client wait time and retention in treatment.

## Findings

Acceptance of Medi-Cal was associated with shorter wait times, whereas organizational readiness for change was positively related to treatment duration. Staff attributes were negatively related to treatment duration. Finally, compared to low program capacity, high program capacity was negatively associated with wait time and positively related to treatment duration.

## Conclusions

Program capacity, an organizational indicator of performance, plays a significant role in access to and duration of treatment. Implications for reducing disparities under the current health-care reform context are discussed.

